# Supplementary material for: Chromothripsis during telomere crisis is independent of NHEJ, and consistent with a replicative origin
Source: Genome Res. 2019 May;29(5):737–49. doi: 10.1101/gr.240705.118 (PMC6499312; doi:10.1101/gr.240705.118)
Supplement: Supplemental Material [file supp_gr.240705.118_Supplemental_file_1.zip › contigs/annotated_contigs/DB73/contig.2.DB73_length_304_mean_cov_6.56907894737.docx]

**DB73_length_304_mean_cov_6.56907894737**

```
TATTATATATATAACAG|AAAATATATATAATATTATATATATAATAATATATATAT|ATAATATGATAAGTTAATATTTTATAATTAA
                  >chr6:48167100-48167141 + E=3e-04 p=1e-03
AAATAAAAAAAAAATATAT|AGTTTTGCTAGTATCTTCATACTTTTATGGATGAGACAGTTTTGGAGATTCTCACTCCATTATTTTCAC
                    >chr3:191696232-191696430 - E=5e-108
TGATGTTCTGCATCCATCTTTTTTTTAACTTTTATTTTAAGTTCACAGGTACATGTGGAAGTTTGTTACATAGGTAAACTTGTGTCATG

GGACTTTGTTGTACAGATTATTTCATCATCCAGGTATTAA
```
